# Assessing Cerebrovascular Resistance in Patients With Sickle Cell Disease

**DOI:** 10.3389/fphys.2022.847969

**Published:** 2022-03-29

**Authors:** Ece Su Sayin, Olivia Sobczyk, Julien Poublanc, David J. Mikulis, Joseph A. Fisher, Kevin H. M. Kuo, James Duffin

**Affiliations:** ^1^ Department of Physiology, University of Toronto, Toronto, ON, Canada; ^2^ Department of Anaesthesia and Pain Management, University Health Network, Toronto, ON, Canada; ^3^ Joint Department of Medical Imaging and the Functional Neuroimaging Laboratory, University Health Network, Toronto, ON, Canada; ^4^ Institute of Medical Sciences, University of Toronto, Toronto, ON, Canada; ^5^ Division of Hematology, Department of Medicine, University of Toronto, Toronto, ON, Canada

**Keywords:** sickle cell disease, cerebrovascular reactivity (CVR), hypercapnic stimulus, magnetic resonance imaging, cerebrovascular resistance

## Abstract

In patients with sickle cell disease (SCD) the delivery of oxygen to the brain is compromised by anemia, abnormal rheology, and steno-occlusive vascular disease. Meeting demands for oxygen delivery requires compensatory features of brain perfusion. The cerebral vasculature’s regulatory function and reserves can be assessed by observing the flow response to a vasoactive stimulus. In a traditional approach we measured voxel-wise change in Blood Oxygen-Level Dependent (BOLD) MRI signal as a surrogate of cerebral blood flow (CBF) in response to a linear progressive ramping of end-tidal partial pressure of carbon dioxide (PETCO_2_). Cerebrovascular reactivity (CVR) was defined as ΔBOLD/ΔPETCO_2_. We used a computer model to fit a virtual sigmoid resistance curve to the progressive CBF response to the stimulus, enabling the calculation of resistance parameters: amplitude, midpoint, range response, resistance sensitivity and vasodilatory reserve. The quality of the resistance sigmoid fit was expressed as the *r* ^2^ of the fit. We tested 35 patients with SCD, as well as 24 healthy subjects to provide an indication of the normal ranges of the resistance parameters. We found that gray matter CVR and resistance amplitude, range, reserve, and sensitivity are reduced in patients with SCD compared to healthy controls, while resistance midpoint was increased. This study is the first to document resistance measures in adult patients with SCD. It is also the first to score these vascular resistance measures in comparison to the normal range. We anticipate these data will complement the current understanding of the cerebral vascular pathophysiology of SCD, identify paths for therapeutic interventions, and provide biomarkers for monitoring the progress of the disease.

## 1 Introduction

Effective regulation of the blood supply to the brain is indicative of the overall health of the cerebral vascular system. Sickle cell disease (SCD), a genetic disorder that causes severe anemia reducing the oxygen carrying capacity of the blood is also associated with disrupted cerebrovascular function (Becklake et al., 1955; Abdu et al., 2008), which remains poorly characterized. In the presence of anemia, brain oxygen delivery is restored by increases in cerebral blood flow (CBF) (Milner, 1974; Herold et al., 1986; Bush et al., 2016). Under the assumption that the maintenance of cerebral metabolic oxygen consumption (CMRO_2_) is prioritized, deficits in brain oxygen delivery result in increases in the oxygen extraction fraction (OEF) at the expense of a reduction in intracellular partial pressure of oxygen (PO_2_). However, it is unknown to what extent the cerebrovascular dysfunction contributes to ischemic injuries often manifesting as silent cerebral infarcts and stroke (Switzer et al., 2006).

**FIGURE 1:**
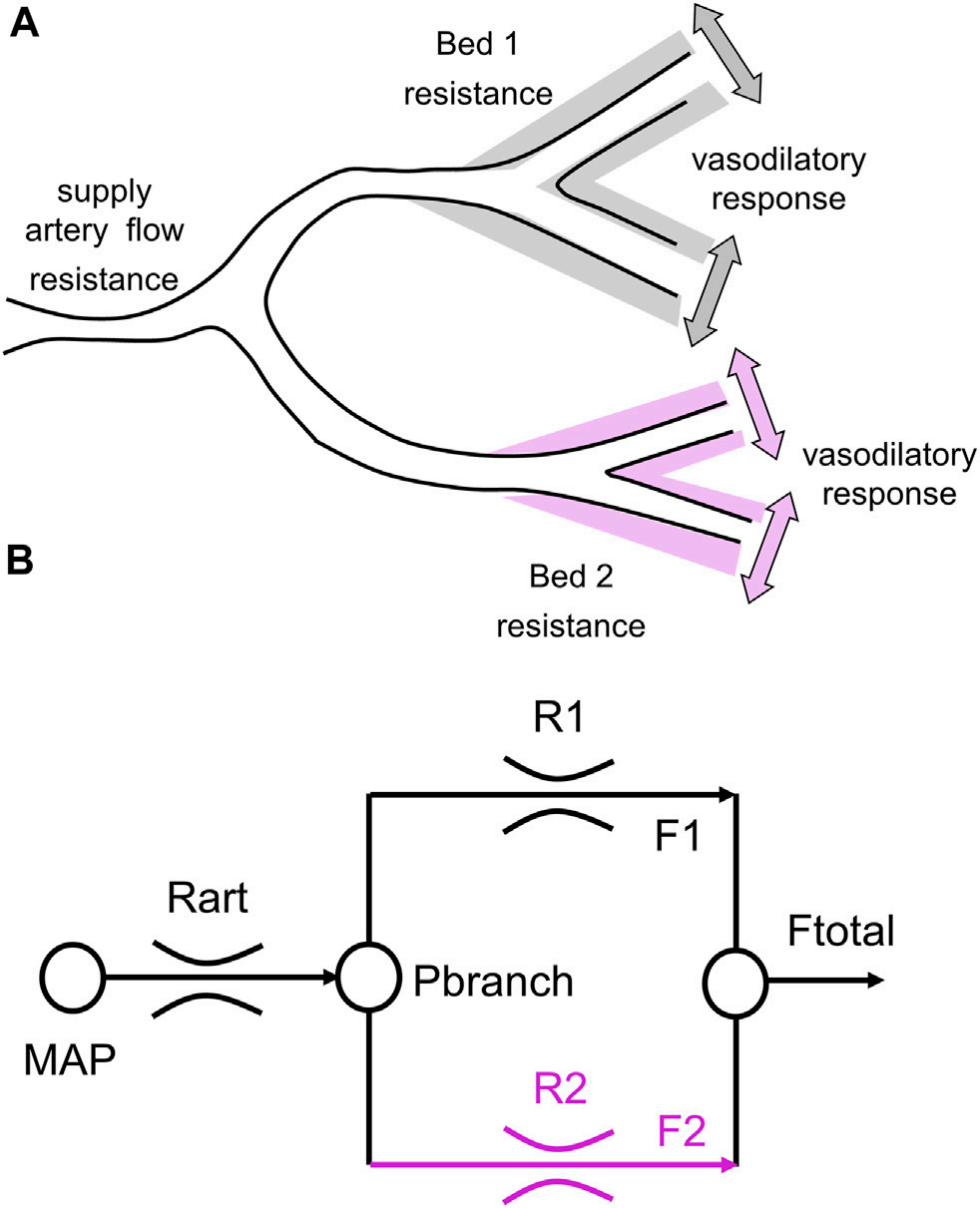
A simple 2-vascular-bed model of a cerebral vascular region. **(A)** A theoretical illustration of two brain vascular territories both supplied in parallel by a major supply artery with resistance. Their vasodilatory responses are shown as shading. **(B)** A simple resistance circuit model. Reference and examined vascular beds with resistances R1 and R2 are perfused via an arterial flow resistance (*Rart*) from mean arterial blood pressure (*MAP*). The pressure perfusing the two branches (Pbranch) and their respective resistances establishes flows through each branch (F1 and F2), that sum to Ftotal. Given F1 and F2 as BOLD measures for the reference and examined voxels, R1 and R2 can be calculated by considering the model as an analog of an electrical circuit (From Duffin et al., 2017).

In patients with SCD, vasodilatory reserve in large vessels is encroached upon by the increase in resting CBF; the threshold for oxygen release from hemoglobin is raised in left-shifted oxyhemoglobin dissociation curve (Abdu et al., 2008); and increased shear stress of anemia and red cell adhesion compromise endothelial cells via nitric oxide-mediated vasodilator activity (Hoiland et al., 2020) and thereby the vasomotor regulation of the microvasculature (Hillery and Panepinto, 2004). These may result in failure of localized hemodynamic compensation for the reduced arterial oxygen content (CaO_2_) of SCD and may account for the observed cognitive deficiencies and widespread ischemic injury in cerebral cortex (Kirk et al., 2009; Pimentel-Coelho et al., 2009) and watershed areas (Hillery and Panepinto, 2004; Fields et al., 2018). We therefore turned our attention to assessing cerebral vascular physiology by observing the patterns and distribution of flow responses to the application of a global vasoactive stimulus (Figure 1A).

A functional assessment of the cerebral vasculature’s regulation of perfusion has been previously tested as the perfusion response to a vasoactive challenge such as acetazolamide (Vaclavu et al., 2017; Vaclavu et al., 2019; Afzali-Hashemi et al., 2021) and hypercapnia (Nur et al., 2009; Prohovnik et al., 2009). The response can be measured in terms of surrogates such as large vessel blood flow velocity measured by trans-cranial Doppler (Nur et al., 2009) or change in Blood Oxygen-Level Dependent (BOLD) signals of magnetic resonance imaging (MRI) (Leung et al., 2016). Early measures of the CBF response to CO_2_ were in terms of a simple regression slope expressed as a ratio of the change in CBF surrogate resulting from a change in the end-tidal partial pressure of CO_2_ (PETCO_2_), termed cerebrovascular reactivity (CVR) (see Fisher and Mikulis (2021) for historic review). In health, the reductions in resistance in all vascular beds is balanced resulting in a sigmoidal increase in flow as a function of PETCO_2_ (Bhogal et al., 2015; Bhogal et al., 2016), reasonably well approximated by a linear fit. In the presence of a range of cerebrovascular pathology distributed to multiple vascular beds, cerebral vessels no longer react in a synchronized fashion, making it difficult to extrapolate from CVR to the varying stimulus-response behavior of vascular beds.

**FIGURE 2:**
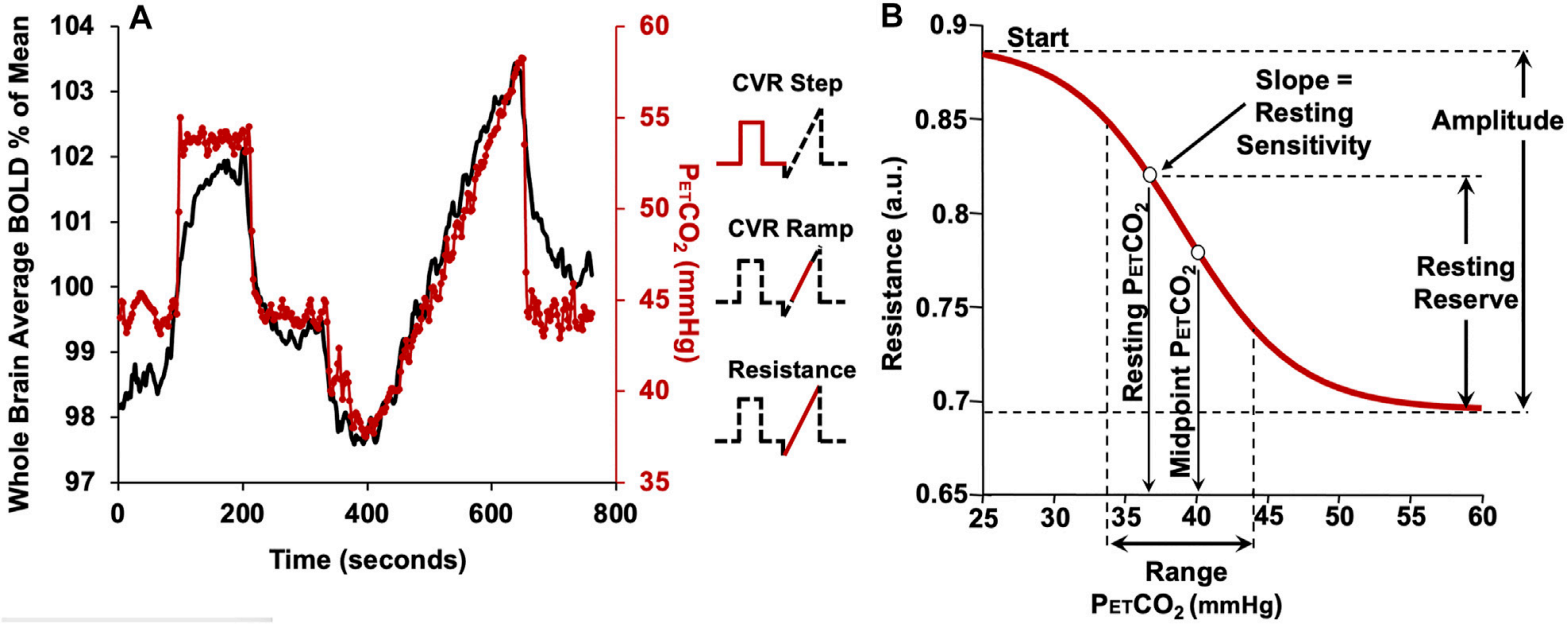
The CVR protocol in a representative subject. **(A)** The CO_2_ stimulus (red line) and whole brain average BOLD response (black line). The partial pressure of CO_2_ (PETCO_2_) is clamped at the subject*’*s resting PETCO_2_ for 2 min, followed by a step increase in PETCO_2_ to 10 mmHg above resting for 2 min*,* followed by a return to the subject’s baseline PETCO_2_ for 2 min. The PETCO_2_ is then reduced by 10 mmHg from the subjects resting level by asking the subject to voluntarily hyperventilate for 1 min, followed by a steady rise in PETCO_2_ (ramp) to 15 mmHg above the resting level over 4.5 min. PETCO_2_ then rapidly returns to baseline for 2 min. The insets show the portion of the protocol used for various analyses. **(B)** The resistance sigmoid and the sigmoid parameters and derived metrics. Start: a relative measure of the vasoconstrictive flow limit in terms of resistance. Amplitude: a measure of the maximum extent of resistance changes. Midpoint: a measure of the displacement of the response with respect to CO_2_. Range: a measure of the CO_2_ range where the resistance response is linear. Sensitivity at resting PETCO_2_: the slope of the straight segment of the sigmoid as an alternate measure of resting state CVR in terms of resistance. Resting reserve: the vasodilatory reserve at resting PETCO_2_ (From Duffin et al., 2017).

In this study we used the ramp portion of the CO_2_ stimulus shown in Figure 2A to produce changes in vascular tone from vasoconstriction to vasodilation so that the full pattern of the flow response may be observed. This is a global stimulus and the high resistance of extracranial portion of cerebral vessels (Faraci et al., 1987) limit the total blood flow capacity of the brain. As the brain’s vascular beds are functionally perfused in parallel, they must compete for the restricted blood supply. In the presence of localized differences in vascular resistance, a rising global vasoactive stimulus results in the disruption of the parallel progressive reduction in resistance (or increase in flow), throughout the brain (Sobczyk et al., 2014; Fisher et al., 2018).

When viewed as a response to a progressive increase in stimulus, as in a linear increase in PETCO_2_, the BOLD response patterns can be classified into four major recognized patterns (Fisher et al., 2017). The vascular beds with the most robust vasodilatory reserve retain a normal appearing sigmoidal pattern of flow. Those beds with little or no vasodilatory reserve have all their flow redistributed to the beds with robust dilatory responses. Those beds retaining a weakened vasodilatory reserve may reduce their resistance at initial vasoactive stimulation but are unable to further reduce resistance at higher CO_2_ levels. Thus, they may increase their flow upon initial increases in CO_2_, but at greater CO_2_ levels reduce their flow in favor of the beds with stronger vasodilatory responses. This results in a biphasic, concave down flow pattern of flow. Some beds display a concave up flow pattern. They behave as if their partial pressure of carbon dioxide (PCO_2_) vs. resistance curve has been shifted to the right: weak reductions in resistance at low PETCO_2_ resulting in steal and rallying reductions in resistance at higher CO_2_ increase flow.

This system has been modeled as an electrical analog shown in Figure 1B. Using this model, all the flow patterns of response can be translated into a single sigmoidal pattern of change in cerebrovascular resistance (Duffin et al., 2018). In this way the various patterns of flow response can be described with a common set of metrics. Figure 2B shows the resistance sigmoid, and the metrics of the fitted equation:

$$R=Start+Amplitude/\left(1+\exp\left(-\left(PETCO_{2}-Midpoint\right)/Range\right)\right)$$

as well as the derived metrics sensitivity and reserve. These five parameters describe the sigmoidal resistance response to CO_2_ and are shown in Figure 2B as amplitude, midpoint, range, resting reserve and resting sensitivity (Duffin et al., 2018; Mcketton et al., 2019).

Here we apply the resistance model approach to assess the functioning of the cerebral vasculature in patients with SCD. The vasoactive stimulus consists of a gradually ramping increase in PETCO_2_, and BOLD MRI signals are taken as surrogates of the CBF response. Maps of regional CVR as well as maps of the resistance analysis parameters were determined for SCD patients. These parameters were also measured in a healthy population to provide an atlas of the normal range of response in terms of the mean and standard deviation in a voxel for each resistance parameter; this enables the generation of z-maps (Sobczyk et al., 2015), a scoring of the patient data in terms of standard deviations from the mean of the normal cohort.

## 2 Methods

### 2.1 Participant and Ethics Approval

This study conformed to the standards set by the latest revision of the Declaration of Helsinki and was approved by the Research Ethics Board of the University Health Network (UHN) and Health Canada. All participants provided written and informed consent to partake in this study. We previously recruited 24 healthy control volunteers with no specific inclusion criteria, referred to as the HC group, between the ages of 18–82 (8F, 35.1 ± 13.8 y median age 30 y and interquartile range is 15.5) by word of mouth and advertisement. The subjects were non-smokers, not on any medication and had no known history of neurological or cardiovascular disease (Sobczyk et al., 2015). For this study we recruited 35 adult patients with SCD (19F, 32.1 ± 13.4 y median age 28 y and interquartile range is 170 (Table 1), through the outpatient haematology clinic at UHN. None of the patients with SCD have previously experienced transfusion therapy. All acquired images were examined for voxels with evidence of white matter hyperintensities and strokes to ensure the selection of participants with no such complications.

**TABLE 1:** Summary of subject demographics.

Age range	HC group	SCD patient group
18–28	10	17
29–38	6	10
39–54	6	5
55–83	2	3
Sex		
F	8	19
M	16	16
Total	24	35

### 2.2 Experimental Protocol

During the entire study, subjects breathed through a mask that was fitted to their face using transparent film (Tegaderm, 3M, Saint Paul, MN, United States) to maintain an airtight seal. Arterial blood gases were controlled using a computerized gas blender following a prospective blood gas targeting algorithm (Slessarev et al., 2007) (RespirAct™, Thornhill Research, Toronto, ON, Canada). While breathing on this system, PETCO_2_ is equivalent to PaCO_2_ (Ito et al., 2008; Willie et al., 2012) enabling highly accurate control of arterial PaCO_2_ targets while maintaining normoxia at the individuals respective resting PETO_2_. The PETCO_2_ protocol and an example of the whole brain BOLD signal response is shown in Figure 2A.

The CVR study was performed on a 3T GE scanner (HDx Signa platform, GE healthcare, Milwaukee, WI, United States) with an 8-channel head coil. The MRI protocol consisted of a high-resolution 3D T1 anatomical whole brain sequence (TI = 450 ms, TR 7.88 ms, TE = 3 ms, flip angle = 12°, voxel size = 0.859 mm × 0.859 mm × 1 mm, matrix size = 256 × 256, 146 slices, field of view = 24 cm × 24 cm, no interslice gap). A standard BOLD MRI echoplanar based gradient echo sequence was then obtained (TR = 2,400 ms, TE = 30 ms, flip angle = 85°, 41 slices, voxel size = 3.5 mm^3^, matrix size = 64 × 64, number of frames = 335, field of view = 24 × 24 cm) while the subject breathed on the RespirAct™.

### 2.3 Data Analysis

MR images and PETCO_2_ data were imported and analyzed using AFNI software (National Institutes of Health, Bethesda, MD, United States) (Cox, 1996). The BOLD images were volume registered, slice-time corrected and co-registered to the anatomical images. The PETCO_2_ data was time-shifted to the point of maximum correlation with the whole brain average BOLD signal which aligns the rapid changes. A linear regression fit of the BOLD signal data to the PETCO_2_ data for the step and ramp portions was then performed on a voxel-by-voxel basis, and the slopes taken as ramp CVR and step CVR. CVR was expressed as the percent change in BOLD signal per change in PETCO_2_ (%/mmHg). The SPGR images (T1 weighted) were segmented into gray matter (GM) and white matter (WM) using SPM8 (Wellcome Department of Imaging Neuroscience, Institute of Neurology, University College, London, United Kingdom). A threshold of 70% probability was applied on the GM and WM maps and then transformed into Montreal Neurological Institute (MNI) space. The ROIs were manually delineated on an anatomical MNI template.

A custom program (LabVIEW, National Instruments, TX, United States) was used to calculate the virtual resistance sigmoid parameters from the ramp data for each voxel. Sigmoid fitting used the Levenberg-Marquardt fitting algorithm. The *r* ^2^ of fit was taken as a quality metric. Since BOLD is a relative measure, the sigmoid start parameter was fixed at 0.75. Fit bounds were −0.001 to −0.3 a.u. for amplitude, 20–60 mmHg for the PETCO_2_ for midpoint and 0.1–10 mmHg PETCO_2_ for range. The choice of boundaries for the fit parameters was based on ranges of values measured in healthy volunteers as described in Duffin et al. (2018). A voxel with any parameters beyond these set bounds, was classified as not fitted and values for this voxel were interpolated from the surrounding voxels values. On average (SD) 18.7% (4.6%) of all voxels for the SCD patient group and 16.7% (3.8%) of all voxels for the HC group were interpolated from surrounding voxels. Resistance maps were generated in original space and converted into MNI space.

The normative data from the 24-person, control group, was co-registered into MNI space using SPM8 aligning corresponding voxels. Mean and SD of CVR and resistance parameters were calculated for each voxel. Atlas map generation is discussed in greater detail in previous publications (Poublanc et al., 2015; Sobczyk et al., 2015; Mcketton et al., 2018). The SCD patient group’s CVR and resistance metrics values were scored by assigning a z-score, the standard deviation from the atlas mean (Sobczyk et al., 2015). The z values were then color coded and overlaid on the anatomical images to create site-specific z-maps. Gray and white matter atlases were created for each of the resistance parameters: amplitude, midpoint, quality, range, reserve and sensitivity. The participant specific GM and WM masks generated previously were used to calculate GM and WM values for CVR and six resistance parameters.

### 2.4 Statistical Analysis

Statistical comparison between HC and SCD patient group ages were made using a one-way analysis of variance (ANOVA) on ranks (Kruskal-Wallis), which determined no significant difference between the age of the two groups (*p* = 0.2). The percentage of interpolated voxels for the resistance sigmoid fitting was also examined using a one-way analysis of variance (ANOVA) on ranks (Kruskal-Wallis), and found no significant difference between the age of the two groups (*p* = 0.071). Similar methods were used to compare the PCO_2_ for GM midpoints, WM midpoints and resting PETCO_2_ using a two-way ANOVA with factors type (GM, WM, resting) and subject (SCD patient, HC). All pairwise multiple comparison procedures (Holm-Sidak method) were used to determine significant differences. Statistical comparisons between all resistance parameters in the HC and SCD patient groups were made using a three-way ANOVA with factors (GM, WM), regions of interest (MCA, PCA, ACA) and group (SCD patient, HC). A Normality test (Shapiro-Wilk) failed for all parameters except for resistance range. Regions of interest in WM and GM were considered significantly different if *p* < 0.05.

## 3 Results

### 3.1 Partial Pressure of Carbon Dioxide Data

The resting PETCO_2_ values and the mean PCO_2_ midpoints for GM and WM are shown in Table 2.

**TABLE 2:** Mean midpoint PCO_2_ values and mean resting PETCO_2_ values compared between SCD patient and HC groups.

	Midpoint (a.u.)		Resting PETCO_2_ (mmHg)
	WM	GM	
SCD patient group	40.3 (3.1)	34.8 (3.5)	41.4 (4.0)
HC group	39.3 (1.8)	33.0 (2.1)	39.6 (3.3)
Comparisons for factor: SCD patient and HC groups			
Comparison	Diff of Means	*p*	*p* < 0.050
SCD patient group vs. HC group	1.453	0.003	Yes
Comparisons for factor: GM/WM/Resting PCO_2_			
Comparison	Diff of Means	*p*	*p* < 0.050
Rest vs. GM	6.605	*p* < 0.001	Yes
WM vs. GM	5.736	*p* < 0.001	Yes
WM vs. Rest	0.869	0.146	No

### 3.2 Atlas of Cerebrovascular Reactivity and Resistance Parameters

**FIGURE 3:**
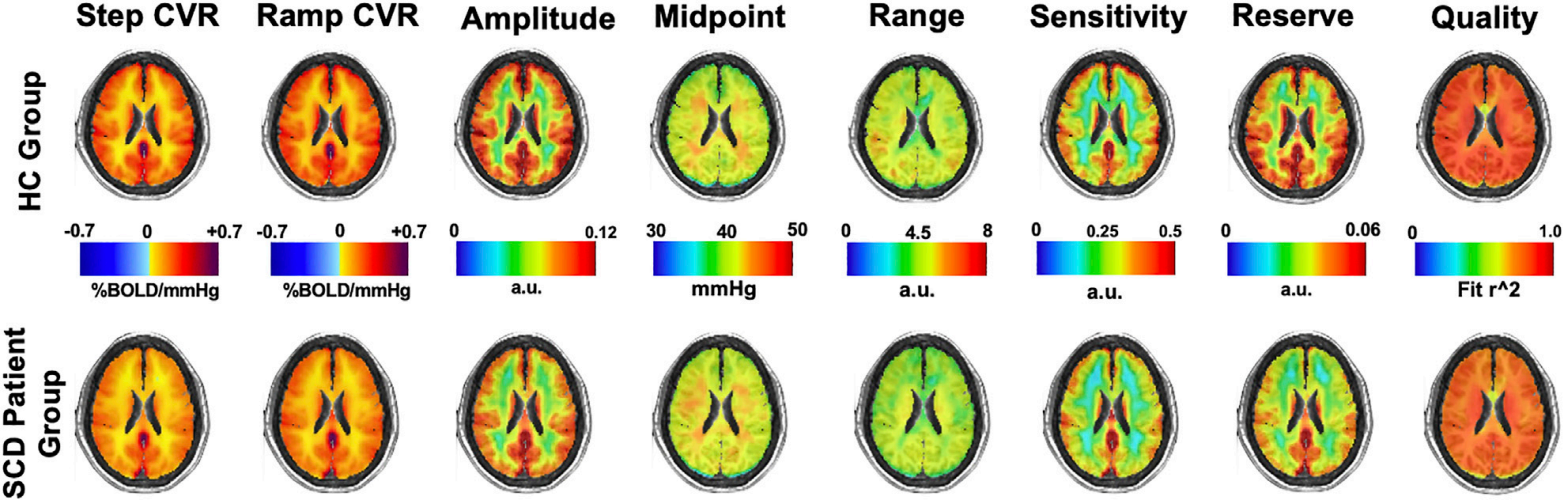
Mean CVR (two left images) and resistance parameters (remaining six images) for the HC and SCD patient groups.

Mean maps of CVR and resistance parameters for the HC group and SCD patient group are shown in Figure 3.

### 3.3 Resistance Parameters Separated Into Vascular Territory ROIs

The CVR and resistance parameters for the healthy control and SCD patient group were calculated for both the WM (Table 3) and GM (Table 4) using vascular territories as regions of interest (ROI): MCA, ACA, PCA. Step CVR was lower in SCD patient group in the MCA, ACA and PCA territories in both WM and GM. CVR measured from the ramp was lower for patients with SCD in all WM and GM territories. Furthermore, resistance amplitude and range were lower in patients with SCD in the WM and GM for all territories. Patients with SCD had decreased resistance reserve in all vascular territories in both WM and GM. The sensitivity did not differ between the two groups in WM for any territory and was slightly reduced for all territories in GM. In all the resistance sigmoid fitting the *r* ^2^ quality exceeded 0.73 and was greater in HC group than the SCD patient group.

**TABLE 3:** The average white matter (WM) CVR and resistance parameters in HC and SCD patient groups for the middle cerebral artery (MCA), anterior cerebral artery (ACA) and posterior cerebral artery (PCA).

	WM					
	HC group			SCD patient group		
	MCA	ACA	PCA	MCA	ACA	PCA
Step CVR (%/mmHg)	**0.094 (0.023)**	**0.063 (0.018)**	**0.162 (0.04)**	**0.081 (0.026)**	**0.05 (0.029)**	**0.137 (0.039)**
Ramp CVR (%/mmHg)	**0.118 (0.024)**	**0.089 (0.027)**	**0.186 (0.022)**	**0.096 (0.037)**	**0.072 (0.041)**	**0.141 (0.045)**
Amplitude (a.u.)	0.065 (0.013)	0.06 (0.012)	0.083 (0.013)	0.061 (0.018)	0.054 (0.015)	0.081 (0.023)
Midpoint (mmHg)	**41.8 (1.8)**	**41.3 (1.9)**	**41.3 (2.4)**	**42.1 (2.6)**	**41.6 (2.2)**	**41.9 (2.9)**
Range (a.u.)	**4.2 (0.6)**	**3.9 (0.6)**	**3.9 (0.6)**	**3.9 (0.7)**	**3.7 (0.7)**	**3.7 (0.7)**
Reserve (a.u.)	**0.033 (0.009)**	**0.029 (0.011)**	**0.044 (0.012)**	**0.023 (0.009)**	**0.024 (0.008)**	**0.039 (0.011)**
Sensitivity (a.u.)	0.218 (0.033)	0.191 (0.033)	0.295 (0.05)	0.22 (0.04)	0.19 (0.05)	0.29 (0.05)
Quality	**0.833 (0.05)**	**0.804 (0.061)**	**0.806 (0.056)**	**0.784 (0.093)**	**0.752 (0.114)**	**0.737 (0.088)**

Regions of interest with significant difference (p < 0.001) between the SCD group and the healthy control are shown in bold.

**TABLE 4:** The average gray matter (GM) CVR and resistance parameters in HC and SCD patient groups for the middle cerebral artery (MCA), anterior cerebral artery (ACA) and posterior cerebral artery (PCA).

	GM					
	HC group			SCD patient group		
	MCA	ACA	PCA	MCA	ACA	PCA
Step CVR (%/mmHg)	**0.253 (0.046)**	**0.235 (0.051)**	**0.36 (0.068)**	**0.178 (0.049)**	**0.153 (0.056)**	**0.259 (0.074)**
Ramp CVR (%/mmHg)	**0.274 (0.037)**	**0.269 (0.047)**	**0.382 (0.049)**	**0.2 (0.054)**	**0.188 (0.066)**	**0.274 (0.079)**
Amplitude (a.u.)	**0.104 (0.015)**	**0.103 (0.015)**	**0.12 (0.016)**	**0.087 (0.018)**	**0.083 (0.019)**	**0.111 (0.024)**
Midpoint (mmHg)	**39.7 (2.3)**	**39.6 (2.4)**	**40.2 (2.9)**	**40.7 (2.7)**	**40.6 (2.6)**	**41.2 (3.1)**
Range (a.u.)	**4.4 (0.6)**	**4.3 (0.5)**	**4.0 (0.60)**	**3.8 (0.6)**	**3.7 (0.7)**	**3.7 (0.7)**
Reserve (a.u.)	**0.048 (0.011)**	**0.046 (0.014)**	**0.064 (0.016)**	**0.039 (0.010)**	**0.036 (0.009)**	**0.057 (0.015)**
Sensitivity (a.u.)	**0.373 (0.051)**	**0.37 (0.064)**	**0.491 (0.091)**	**0.34 (0.067)**	**0.33 (0.081)**	**0.46 (0.098)**
Quality	**0.84 (0.044)**	**0.84 (0.046)**	**0.806 (0.056)**	**0.776 (0.074)**	**0.773 (0.087)**	**0.751 (0.077)**

Regions of interest with significant difference (p < 0.001) between the SCD patient group and the healthy control are shown in bold.

**FIGURE 4:**
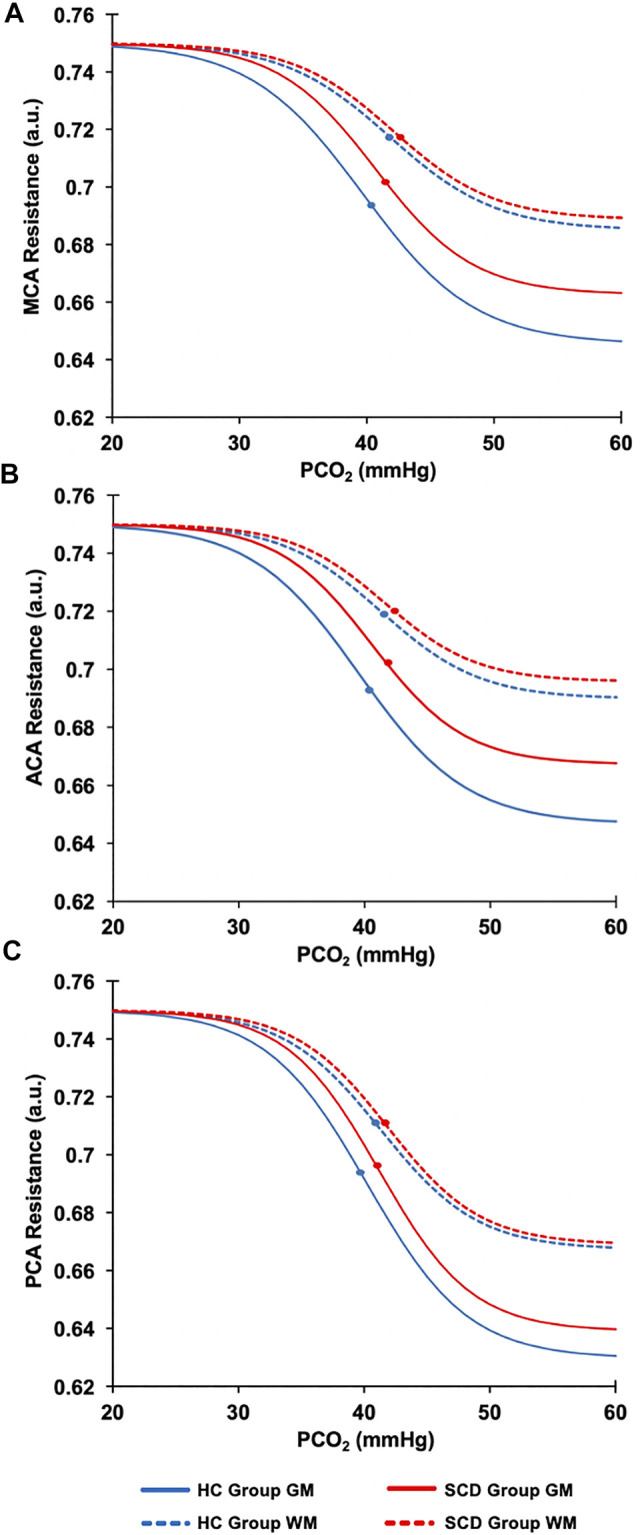
The resistance sigmoidal curves for the HC (blue line) and the SCD patient (red line) groups in GM (solid lines) and WM (dashed lines) calculated from the mean values of the resistance sigmoid parameters. **(A)** The MCA sigmoid curves for the GM and WM for HC and SCD patient groups. **(B)** The ACA sigmoid curves for the GM and WM for HC and SCD patient groups. **(C)** The PCA sigmoid curves for the GM and WM for HC and SCD patient groups. The dots on each sigmoid curve represent the resistance reserve (from the dot to the lowest resistance).

The resistance sigmoid parameters, amplitude, midpoint and range were used to create sigmoids for GM and WM in each vascular territory for the healthy control group and SCD patient group assuming the same start value for all of 0.75 a.u. (Figures 4A–C).

**FIGURE 5:**
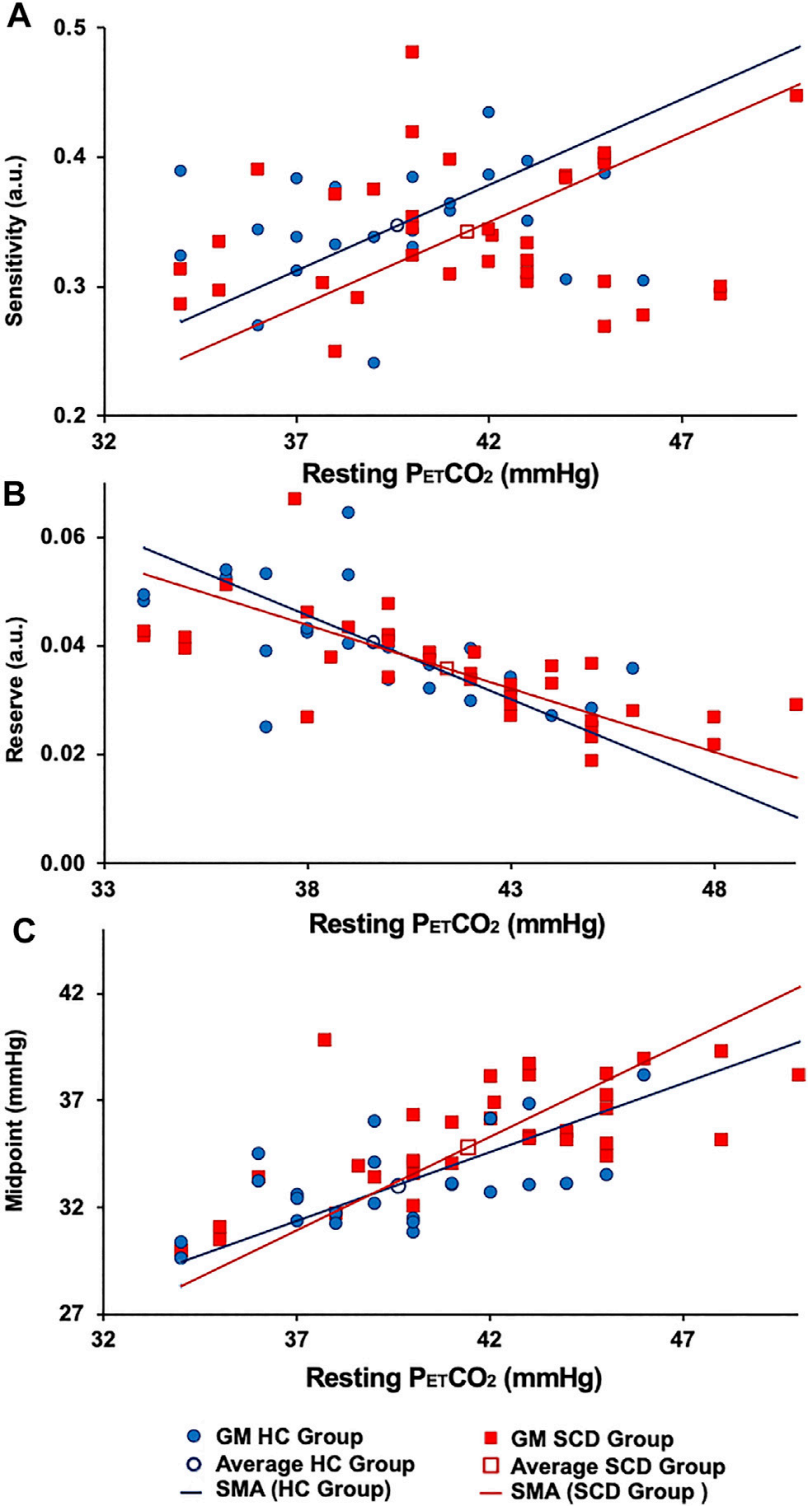
The **(A)** resting resistance sensitivity, **(B)** resting resistance reserve. **(C)** Resting resistance midpoint in GM HC (blue lines) and GM SCD (red lines). Note: the difference in the average resting midpoints between the two groups.

Sigmoid resistance curves were also calculated based on the average sigmoid parameters in GM for the whole brain and from these the reserve and sensitivity were determined. Figure 5 shows the relations between these measures and the resting PETCO_2_ for all participants.

### 3.4 Cerebrovascular Reactivity and Resistance Maps and Z-Maps

**FIGURE 6:**
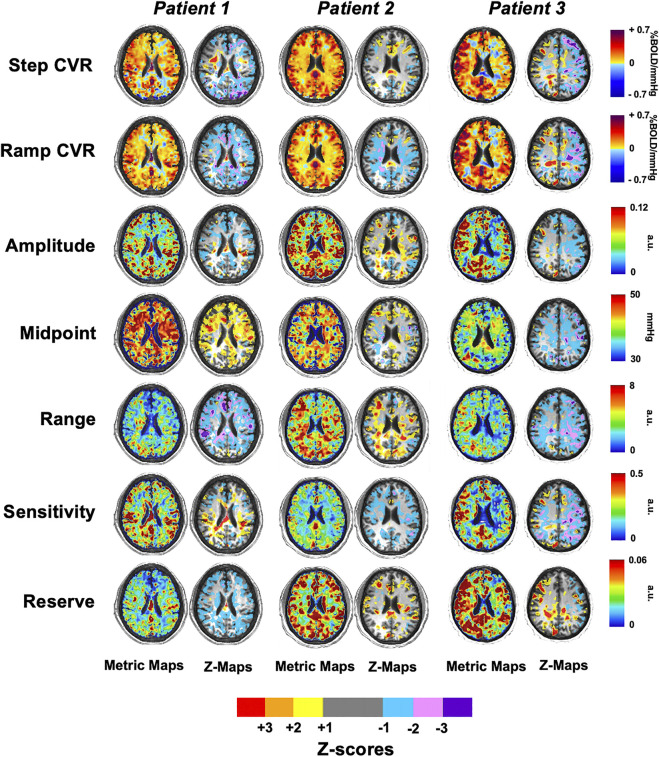
Axial slices of three representative patients with SCD for the CVR and five resistance parameters that were z-scored against the healthy control atlas.

Figure 6 shows single axial images for the CVR and five resistance parameters which were generated for three patients with SCD and z-scored against the HC group (atlas) as previously shown in Figure 2. The areas of significant difference in WM and GM between the two groups are shown in their respective z-maps.

## 4 Discussion

Cerebral blood flow is regulated over a range of perfusion pressures by adjusting cerebrovascular resistance with the constriction and dilation of arterioles (Klein et al., 2021). Patients or individuals with sickle cell disease compensate for their anemia by increasing CBF (Milner, 1974; Bush et al., 2016), achieved not only by vasodilation but also by an increase in the diameter of major intracranial and extracranial vessels (Guilliams et al., 2020). The main finding of this study is that the functional regulation of the cerebral vasculature of patients with SCD is impaired compared to healthy controls. Detailed findings are summarised in Tables 3, 4 with significant differences for GM and WM in all regions.

Cerebrovascular reactivity for both step and ramp CO_2_ challenges is decreased in WM and GM for all ROIs, and the resistance sigmoid parameter resting vasodilatory reserve is decreased in both GM and WM in all vascular territories. These differences show that the ability to increase CBF in response to a challenge is decreased in SCD patients compared to healthy controls. Other parameters of the resistance sigmoids in the SCD patient group are also different from those of healthy controls. These include a reduced resistance sigmoid amplitude in GM, indicating that the full extent of vasodilation and vasoconstriction is restricted in SCD patients. The resistance resting sensitivity is also reduced so that the vasodilatory response to changes in PCO_2_ from resting state is less. Moreover, the resistance sigmoid midpoint, the PCO_2_ about which vascular changes occur, is elevated in SCD patients. This midpoint finding has implications for cerebrovascular regulation, which are discussed below.

This is the first study to measure the resistance parameters in patients with SCD. Previous studies, which examined the effects of ageing, where some aspects of vascular resistance in parts of the brain were found to be initially maintained with age but then increased in later years (Mcketton et al., 2019), also showed significant reductions in resistance sigmoid parameters in mild cognitive impairment. The same study demonstrated that the resistance measures provided physiological explanations for the various observed types of BOLD-CO_2_ response patterns (Duffin et al., 2017). Here we present the first measures of resistance sigmoid parameters in response to a progressive vasoactive stimulus as a manifestation of the pathology in patients with SCD accumulated over childhood and early adulthood. As such we do not have data as to the state of resistance responses of cerebral vessels in SCD at early ages or the rate and pattern of the development of the pathology we observed. However, in this data, one may identify candidate biomarkers of disease progression, prognosis and therapeutic targets.

The virtual resistance model analysis of the CBF response to a ramp change of PETCO_2_ was examined in Duffin et al. (2017), and has been described in detail in Duffin et al. (2018). In brief, the observed local (voxel) BOLD signal responses to a global CO_2_ stimulus are the result of both changes in local perfusion pressure caused by network competition for a CBF supply limited by the high resistance of the major supply vessels (Faraci and Heistad, 1990), and changes in vessel diameter and therefore vessel flow resistance with CO_2_ (Duffin et al., 2021). These factors cannot be separated. However, using a virtual reference resistance in one branch of the model (Figure 1) to compete with an examined voxel’s BOLD response to a ramp of CO_2_ can provide a simulated network interaction change in perfusion pressure. The actual reference resistance competition for each voxel is likely different and at this point, unknowable. Consequently, the model only calculates a virtual resistance variation with CO_2_ in the examined voxel that would produce the observed BOLD response if it was in competition with the reference, not the actual resistance variation with CO_2_. In other words, if the voxel blood flow resulted from competing with the reference resistance, the model calculates the corresponding resistance variation with CO_2_.

By making the reference resistance response to CO_2_ the same for all observed voxel BOLD signal response to CO_2_, the resistance pattern of variation with CO_2_ can be compared between voxels because it is entirely determined by the measured BOLD response to CO_2_. The model simply converts the BOLD signal variation with the global CO_2_ stimulus into a more physiologically meaningful sigmoidal resistance form, where the parameters of the resistance sigmoid provide a description of the voxel BOLD response to a global CO_2_ stimulus. The resistance sigmoid is still a result of both vasodilation in response to changes in PCO_2_ and vascular network pressure changes, as reflected in changes in the BOLD signal.

Figure 4 shows the regional sigmoids for the SCD and HC groups in both GM and WM. The change in resistance between that indicated by the dots on each sigmoid and the minimum resistance illustrate the resting resistance reserve calculated from the regional averages. The figure illustrates the findings of Tables 3, 4 that resting resistance reserve is reduced in SCD patients compared to healthy controls, and that the resistance sigmoid midpoint PETCO_2_ values for the SCD patients are higher. These two observations are linked to differences in the resting PETCO_2_, which is a little higher in SCD patients (Table 2). As Figure 5 shows, resting resistance reserves and resistance sigmoid midpoints are proportionate to resting PETCO_2_, while resting resistance sensitivities are not as strongly linked. We suggest the following physiological explanation.

In a model we proposed for the smooth muscle regulation of cerebral blood flow (Duffin et al., 2021), vessel tone was related to intracellular [H^+^], which was regulated to a value commensurate with normal intracellular function. Increases in arterial PCO_2_ elevate the smooth muscle intracellular [H^+^], and, although intracellular acid-base regulation attempts to return [H^+^] to normal, restoration is not complete, vascular tone relaxes, and cerebral blood flow increases. When ramp increases in arterial PCO_2_ are employed as a vasodilatory stimulus, smooth muscle intracellular [H^+^] increases and resistance decreases progressively to increase CBF. This decrease in resistance consumes vasodilatory reserve as measured by the resting resistance reserve. Indeed, as Figure 5 shows there is a proportionate decrease in reserve with increased resting PETCO_2_. In order to restore the reserve, the sigmoid resistance relation must be right shifted to a higher PCO_2_, as measured by an increase in the midpoint PCO_2_. That this adaptation occurs is shown in Figure 5 where there is a proportionate increase in resistance sigmoid midpoints with increased resting PETCO_2_.

Although in all the resistance sigmoid fitting the *r* ^2^ quality exceeded 0.7, the quality measure was lower in the WM and GM of the patients with SCD compared to the HC group. The reasons for this difference in quality are unknown. The percent of interpolated voxels was not significantly different between the groups, and so we do not think this aspect of the analysis was responsible. Whether the difference was due to differences between the groups in the MR acquisition (e.g., motion artifact) or some physiological aspect (cerebrovascular pathology) cannot be determined. A low quality measure in a voxel reflects a low signal to noise ratio and so quality maps are used to identify regions where the resistance measures are less reliable for making conclusions.

## 5 Limitations

The hemoglobinopathies in the SCD patient group varied (29 HbSS, 5 HbSC and 1 HbSD). Since it is uncertain how the BOLD response is affected for specific hemoglobinopathies we continue to interpret BOLD responses in the same way as established with HbA, but this possible confounder should be noted. Furthermore, treatments such as hydroxyurea, analgesics, and the development of other related and unrelated comorbidities and their treatment are added diversifying factors. These many differences within our SCD patient population could impose a degree of variability for which we could not control in such a small cohort, and thus certainly contributed to the obscuration of some of the characteristics of the disease. We also suggest that, because we studied adults rather than children, the distinguishing features of SCD had more time to be established, making them easier to detect in comparison to the healthy cohort. However, our study is biased toward the selection of healthier SCD patients, so that the differences in their resistance parameters from those of the healthy group are an indication of the effects of SCD without the confounding effects of other factor such as the presence of silent infarcts. The characteristics showing statistical significance should be appreciated in this regard.

## 6 Conclusion

Cerebrovascular reserve is reduced in patients with SCD compared to healthy controls as evidenced by decreased CVR and decreased resistance reserve at resting PETCO_2_, so that these individuals unable to respond as well to a stress as healthy individuals. Moreover, the extent to which cerebrovascular resistance can be adjusted is also compromised, with a reduced resistance sigmoid amplitude. However, the mechanism of adaptation to a generally higher resting PETCO_2_ appears similar to that of healthy controls as shown by the increase in resistance sigmoid midpoint.

## Data Availability

The original contributions presented in the study are included in the article/Supplementary Material, further inquiries can be directed to the corresponding author.
